# Intestinal parasite prevalences in dogs and cats: a decade of retrospective data from a reference veterinary laboratory in Madrid, Spain

**DOI:** 10.1186/s13071-025-07168-1

**Published:** 2025-12-18

**Authors:** J. P. Barrera, A. Montoya, V. Marino, J. Sarquis, R. Checa, D. Carmena, E. Estévez-Sánchez, C. Gómez-Velasco, P. Moraleda, L. Cano, I. Fuentes, G. Miró

**Affiliations:** 1 PetParasiteLab, Department of Animal Health, Veterinary Faculty, Universidad Complutense de Madrid, Avda. Puerta de Hierro s/n, 28040 Madrid, Spain; 2 Department of Microbiology and Parasitology, Pharmacy Faculty, Universidad Complutense de Madrid. Pl. de Ramón y Cajal s/n, Madrid, Spain; 3 Parasitology Reference and Research Laboratory, Spanish National Centre for Microbiology, Instituto de Salud Carlos III, Majadahonda, Madrid, Spain

**Keywords:** Intestinal parasite, Dog, Cat, Coprological analysis, Diagnosis

## Abstract

**Background:**

Spain’s recent abrupt rise in numbers of registered pet dogs and cats has intensified the need for proper animal health care, as 60% of infectious diseases are zoonotic. While pathogen detection has improved through advances in molecular techniques, pet owners often fail to adhere to veterinary guidelines, increasing infection risks. Among the diagnostic tools available, faecal analysis plays a key role in detecting zoonotic parasites such as *Giardia duodenalis*, *Cryptosporidium* spp. and *Toxocara* spp. This study was designed to assess intestinal parasite prevalence in dogs and cats along with epidemiological trends.

**Methods:**

Between 2013 and 2023, a total of 15,899 faecal samples from dogs and cats submitted to a reference laboratory of parasitology in Madrid (Spain) were analysed using Mini-FLOTAC^®^, merthiolate–iodine–formalin (MIF), Baermann–Wetzel (for lungworms and *S. stercoralis* when indicated) and direct immunofluorescence assays, with molecular confirmation by polymerase chain reaction (PCR) when required. Epidemiological variables were statistically analysed.

**Results:**

Overall, 26% of dogs and 21.4% of cats tested positive for at least one parasite. Protozoan infections were more prevalent overall, particularly *G. duodenalis* in dogs (16.0%) and *Cystoisospora* spp. in cats (7.8%). In contrast, helminth infections such as *T. cati* (7.6%) were more frequent in cats than in dogs. Dogs were more commonly infected by protozoa than helminths, while cats showed a more balanced distribution between both groups. Co-infections occurred in 13.6% of positive samples, with *G. duodenalis* being frequently involved.

Puppies and kittens were more susceptible to infection, likely owing to an immature immune system. In animals from shelters, infection rates were higher than in owned pets. Seasonal variations were clearly observed, such that *G. duodenalis* peaked in winter and helminths in autumn. Over time, the prevalence of *G. duodenalis* increased, while that of *Cystoisospora* spp. declined.

**Conclusions:**

These findings highlight the importance of parasite control for purposes of both animal and public health, and emphasize a need for regular faecal testing, deworming and improving owner awareness of parasites. To minimize zoonotic risks and improve pet health management, we would recommend standardizing diagnostic procedures and designing suitable veterinary interventions.

**Graphical Abstract:**

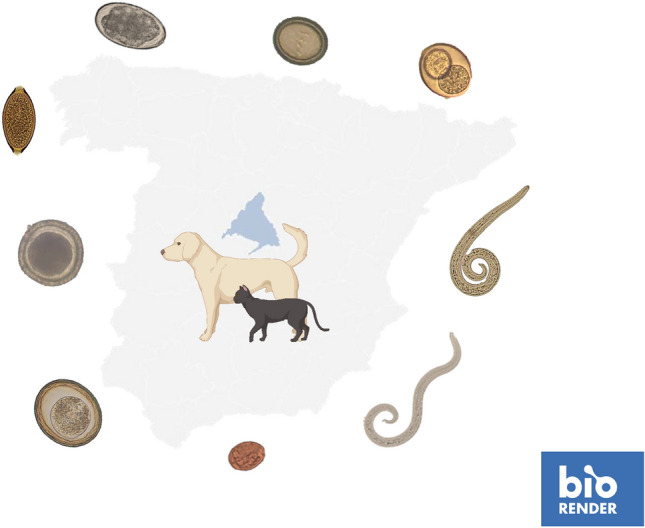

## Background

Global figures of companion animals have shown a steady increase, especially since the 2019 coronavirus disease (COVID-19) pandemic. For instance, according to data published in 2023 [1], Spain had 9.3 million and 5.8 million registered dogs and cats, respectively, notably exceeding the population of children under the age of 15 years. This rising trend has been attributed to factors such as remote work, more time spent at home and changes in leisure activities during the pandemic [1]. Consequently, proper health check-ups in pets are crucial, as 60% of infectious diseases are zoonotic [2]. Advances in molecular techniques have improved the detection of pathogens, which can now be identified with greater precision [3].

Advances have been made both in the diagnosis of diseases in pets and in prevention, particularly through the implementation of healthcare plans that reinforce early diagnosis in small animal practices [4]. However, owners do not always adhere strictly to veterinarian’s recommendations, thereby increasing the risk of infection for both pets and people [5]. Among the tests usually included in healthcare plans, a coprological examination should always be undertaken owing to the ease and non-invasive nature of sample collection and the significance of the diagnosis obtained, as among the detectable infectious agents that affect dogs and cats, several zoonotic agents can be detected [6–9].

Faecal analysis is a routine procedure in many veterinary practices. However, it is often performed without sufficient attention paid to proper methodologies. While coprological tests are best performed by veterinarians or experienced veterinary technicians, this task is often delegated to the less experienced team members, usually with minimal instruction or emphasis on its significance [10]. The need should, therefore, be stressed to submit faecal samples to specialized veterinary reference parasitology laboratories, where staff are properly trained in these procedures.

Among the most relevant parasites that can be found in dog and cat faeces in Spain and other Mediterranean countries are protozoa (e.g. *Giardia duodenalis*, *Cryptosporidium* spp. and *Cystoisospora* spp.), nematodes (e.g. *Toxocara canis*, *Toxocara cati*, *Toxascaris leonina*, the Ancylostomatidae family, *Trichuris vulpis*, *Eucoleus aerophilus* and *Strongyloides stercoralis*) and cestodes (e.g. the Taeniidae family, *Dipylidium caninum* and *Joyeuxiella* spp.). In addition, some bronchopulmonary nematodes can be detected in faecal samples from carnivores (e.g. *Angiostrongylus vasorum*, *Aelurostrongylus abstrusus* and *Troglostrongylus* spp.) [11].

In general, animals infected with intestinal parasites show a wide range of clinical signs, ranging from subclinical infections, moderate to severe diarrhoea or, in extreme cases, even death [12]. Migrating parasites (such as *Toxocara* spp. and *Ancylostoma* spp.) are particularly worrying, as massive parasitism in animals under 1 year of age can lead to verminous pneumonia or intestinal intussusception [13]. Similarly, some of the zoonotic parasites mentioned can cause a similar range of clinical signs in humans, with more severe symptoms occurring in vulnerable populations, such as children aged under 5 years, the older population and immunocompromised individuals [6]. This is particularly true for infections caused by *G. duodenalis* and *Cryptosporidium* spp. Migratory parasites can lead to conditions such as cutaneous larva migrans (ancylostomids), larva currens (*S. stercoralis*) and ocular and visceral larva migrans (*T. canis* and *T. cati*) [14]. This highlights a need to detect intestinal and/or respiratory parasites, not only because of their impacts on public and animal health but also because prompt treatment of animals will prevent the environmental infection [15].

In the present study, we examined data derived from different coprological methods used on canine and feline faeces samples submitted to a veterinary parasitology reference laboratory by veterinary practices, breeding sites, stray cats and animal protection shelters located in the Community of Madrid (Spain). Our aims were to assess the prevalence of the most common intestinal parasite infections, to explore their potential associations with epidemiological variables and to examine trends over a 10-year period in dogs and cats.

## Methods

### Study design and sample collection

In this retrospective study, faecal samples from dogs and cats collected from 2013 to 2023 were analysed at PetParasiteLab, a reference laboratory for the diagnosis of infectious diseases in carnivores (Veterinary Faculty, Universidad Complutense de Madrid, Madrid, Spain). Only faecal samples from animals not previously registered as patients in the laboratory were included, corresponding to their first coprological analysis. These analyses were performed mainly as part of annual health check-ups covered by pet insurance, during the introduction of new animals into shelters or as control measures in cat colonies. This criterion was applied to avoid potential bias from prior administration of antiparasitic treatments that could alter true prevalence estimates. Samples collected during post-treatment follow-up were excluded, as persistent cyst excretion after effective therapy (e.g. in *G. duodenalis* infections) could lead to an overestimation of prevalence.

Veterinarians completed a clinical form (CRF) and submitted the faecal samples. Three consecutive daily samples per animal were analysed whenever available; in cases where this was not possible, a single sample was examined. Every CRF included the signalment of each animal, and the variables analysed were species (dog/cat), age (< 1 year/≥ 1 year), sex (male/female), origin (breeding dog/stray cats, owner and shelter), season (winter, spring, summer and autumn) and faecal consistency. The latter was determined by laboratory personnel using the Bristol Stool Chart (1–7) [16, 17], where a score of 1 corresponded to hard/dry stools and 7 indicated liquid diarrhoea.

### Routine laboratory protocol for coprological analysis

Faecal samples were analysed using different techniques, depending on the type of coprological analysis requested, following internal validated standard operating procedures (SOPs). Samples were first macroscopically inspected. For the coprological analysis, Mini-FLOTAC^®^ was used to detect oocysts, eggs and/or larvae of the main intestinal parasites, and the merthiolate–iodine–formalin (MIF) method was carried out to detect *G. duodenalis* cysts.

When requested, an immunodiagnostic test was performed by direct immunofluorescence assay (DFA) to detect cysts of *G. duodenalis* and oocysts of *Cryptosporidium* spp. along with a molecular diagnosis to detect infection by *G. duodenalis*, *Toxoplasma gondii*, *S. stercoralis* (real-time polymerase chain reaction [PCR]) and *Cryptosporidium* spp. (conventional nested PCR). A *G. duodenalis* infection was considered present when at least one positive result was obtained through the MIF and/or DFA methods. Likewise, *Cryptosporidium* infection was recorded on the basis of a positive DFA result. Molecular techniques for *G. duodenalis* and *Cryptosporidium* spp. were employed only for confirmation and diagnostic support and not as the main diagnostic method.

The Baermann–Wetzel method was also used to detect lungworms and *S. stercoralis* when there was clinical suspicion on the basis of clinical signs such as coughing and/or lifestyle factors (e.g. habitat, aptitude, etc.). In consequence, the number of samples analysed with this technique was lower.

### Flotation method (Mini-FLOTAC^®^)

The flotation method requiring a Mini-FLOTAC^®^ device was used on cat and dog faecal samples according to the manufacturer’s instructions [18, 19].

### Merthiolate–iodine–formalin (MIF) method

**Fig. 1:**
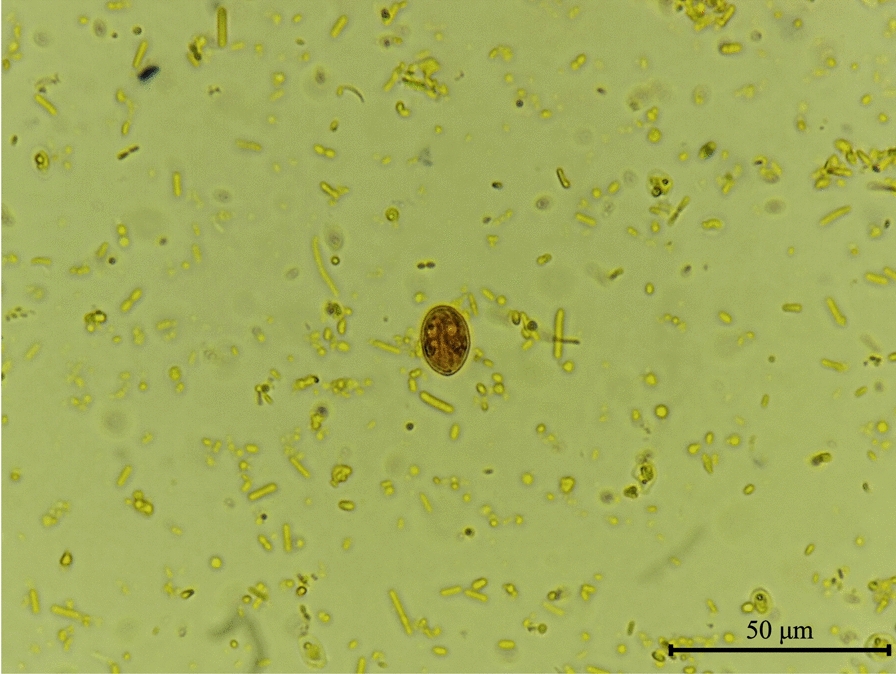
*Giardia duodenalis* cyst (15 × 12 μm) detected by the MIF method. 1000×

This procedure is particularly useful to detect *G. duodenalis* cysts in faecal concentrates prepared from pooled samples collected over 3–5 consecutive days from the host. This method [20] is useful to partially distinguish between active cysts (Fig. 1) and degenerated ones on the basis of their morphology [21].

### Direct immunofluorescence assay (DFA)

**Fig. 2:**
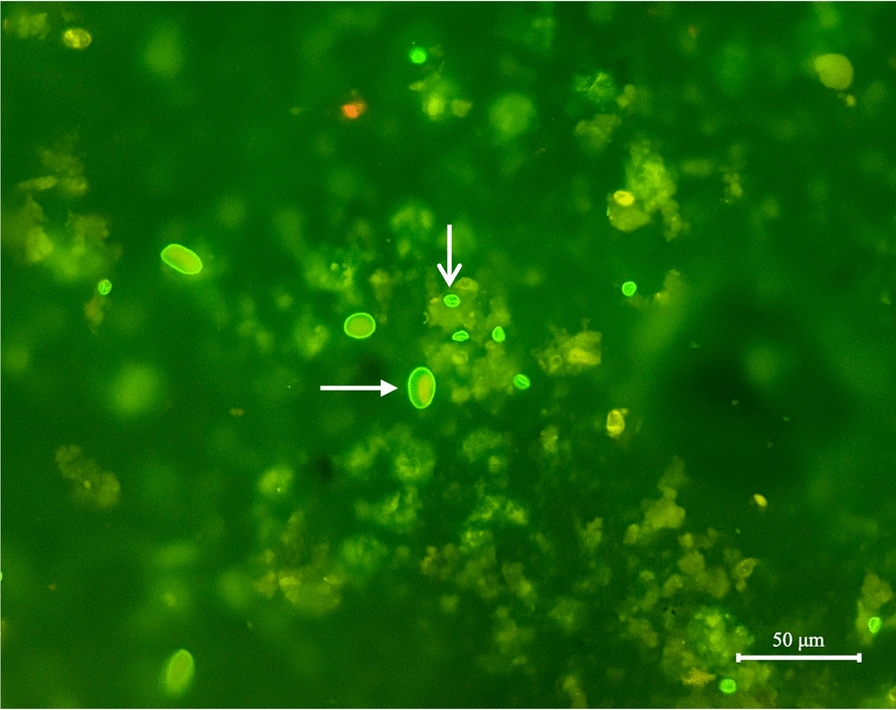
*Giardia duodenalis* cyst (narrow-headed white arrow) and *Cryptosporidium* spp. oocyst (wide-headed white arrow) detected by the DFA method. 400×

The commercial Crypto/Giardia Cel IF^®^ Kit (CeLLabs, Brookvale, Australia) was used following the manufacturer’s manual. The resulting slides were examined under a Nikon Eclipse Ci-S fluorescence microscope (Nikon, Tokyo, Japan) at 400× magnification. Round to oval structures of appropriate size (*G. duodenalis* cysts: 8–12 μm; *Cryptosporidium* oocysts: 4–6 μm) visible as bright apple-green fluorescence were identified as positive (Fig. 2).

### Baermann–Wetzel method

This method relies on the movement of living nematode larvae from faeces into the surrounding water, where they are collected in a funnel. A plastic funnel was attached to a 10-cm-long piece of soft silicone tube, which was sealed with a pinch clamp positioned at an angle to the tube’s tip. Water was added to the funnel together with a surfactant solution, and 5 g of faecal sample was placed on a gauze and wrapped up. The funnel was left at room temperature for 8 h, allowing the larvae to leave the faeces and settle at the tip of the tube by sedimentation. Next, the pinch clamp was opened and the liquid drained into a 10-ml tube. This tube was then centrifuged at 1500 rpm for 10 min. Next, the supernatant was discarded and the sediment examined under a microscope [11]. Pulmonary nematodes and compatible *S. stercoralis* larvae were identified on the basis of keys [22, 23].

### Faecal DNA extraction and purification

DNA was extracted from 200 mg of each concentrated faecal sample using the QIAamp DNA Stool Mini Kit (Qiagen, Hilden, Germany) following the manufacturer’s protocol. The samples, mixed with InhibitEX buffer, were incubated for 10 min at 95 °C. The purified DNA was then eluted in 200 μl of PCR-grade water and stored at 4 °C until further PCR analysis.

### PCR methods

For the detection of *G. duodenalis*, *T. gondii* and *S. stercoralis*, real-time PCR (qPCR) assays were used, targeting highly conserved genes such as *18S* rRNA gene for *G. duodenalis* and *S. stercoralis,* and the *B1* gene for *T. gondii*, allowing for rapid and sensitive identification. For *Cryptosporidium* spp., a nested PCR was performed to amplify a 587 bp fragment of the *18S* rRNA gene, which enhances diagnostic sensitivity in low-load infections. Primer sequences, amplicon sizes, PCR methods and the references are presented in Table 1.

**Table 1:** Oligonucleotides and PCR conditions used for the molecular detection of intestinal parasites

Target gene	Primers (nucleotide sequence 5′-3′)	Amplicon size	Method	Reference
*G. duodenalis* *18S rRNA*	Gd-80F: 5′-GACGGCTCAGGACAACGGTT-3′ Gd-127R: 5′-TTGCCAGCGGTGTCCG-3′	62 bp	Real-time PCR	[24]
*Cryptosporidium* spp. *18S rRNA*	CR-P1: 5′-CAGGGAGGTAGTGACAAGAA-3′ CR-P2: 5′-TCAGCCTTGCGACCATACTC-3′ CR-P3: 5′-ATTGGAGGGCAAGTCTGGTG-3′ CPB-DIAGR: 5′-TAAGGTGCTGAAGGAGTAAGG-3′	652 bp 652 bp	Nested PCR	[25]
*T. gondii* *B1* gene	23-mer 5′-GGAGGACTGGCAACCTGGTGTCG-3′ 25-mer 5′-TTGTTTCACCCGGACCGTTTAGCAG-3′	126 bp	Real-time PCR	[26]
*S. stercoralis* *18S rRNA*	Stro18S-1530F: 5′-GAATTCCAAGTAAACGTAAGTCATTAGC-3′ Stro18S-1630R: 5′-TGCCTCTGGATATTGCTCAGTTC-3′	101 bp	Real-time PCR	[27]

*rRNA* ribosomal ribonucleic acid

*bp* base pair

### Sequencing analyses

PCR products were sequenced in both directions using the corresponding internal primer sets described above, employing BigDye^™^ chemistry and an ABI 3730xl sequencer (Applied Biosystems^®^). Raw sequencing data from both forward and reverse directions were analysed using Chromas Lite version 2.1. Nucleotide sequences were compared with reference sequences from the National Center for Biotechnology Information (NCBI) database using the Basic Local Alignment Search Tool (BLAST) tool. The resulting DNA consensus sequences were aligned with reference sequences using MEGA 11 software to confirm species identity.

Phylogenetic relations between the *Cryptosporidium* sequences identified in this study and those retrieved from the NCBI repository were analysed using the neighbour-joining (NJ) method in MEGA 11 [28, 29]. Genetic distances were calculated with the Kimura 2-parameter model, while rate variation among sites was modelled using a Gamma distribution (shape parameter = 2).

### Statistical analysis

To assess associations between the variables examined and infections by the different species of the parasite, the chi-squared test was employed. All statistical tests were conducted using the SPSS Statistics package version 17.0 (IBM, Chicago, IL, USA). Significance was set at a confidence level of 95% (*P* < 0.05).

## Results

### Prevalence rates

Over a 10-year period (2013–2023), 15,889 faecal samples (10,813 from dogs and 5086 from cats) were analysed along with several epidemiological variables (Table 2). The global prevalence of detected parasites is presented in Table 3. Using previously described coprological methods, 26% (2801/10,813) of the dog samples and 21.4% (1090/5086) of the cat samples tested positive for at least one parasite.

**Table 2:** Distribution of dogs and cats by sex, age, origin, faecal consistency, season and year of the analysis

Variable	Dogs (*n* = 10,813)		Cats (*n* = 5086)		Total (*n* = 15,889)	
	*n*	%	*n*	%	*n*	%
Age						
< 1 year	489	4.5	276	5.4	765	4.8
≥ 1 year	4162	38.5	1053	20.7	5215	32.8
Total	4651		1329		5980	
Sex						
Male	4682	43.3	2223	43.7	6905	43.5
Female	4806	44.4	2357	46.3	7163	45.1
Total	9488		4580		14,068	
Origin						
Breeders/stray cats	2294	21.2	762	15.0	3056	19.2
Owned	2977	27.5	1070	21.0	4047	25.5
Shelters	5399	49.9	3254	64.0	8653	54.5
Total	10,670		5086		15,756	
Faecal consistency						
1–2	61	0.6	194	3.8	255	1.6
3–4	2829	26.2	1414	27.8	4243	26.7
5–7	1884	17.4	329	6.5	2213	13.9
Total	4774		1937	38.1	6711	42.2
Season						
Winter	2674	24.7	1095	21.5	3769	23.7
Spring	2969	27.5	1157	22.7	4126	26.0
Summer	2091	19.3	1438	28.3	3529	22.2
Autumn	3079	28.5	1396	27.4	4475	28.1
Total	10,813		5086		15,899	
Years						
2013	666	6.2	89	1.7	755	4.8
2014	795	7.4	337	6.6	1132	7.2
2015	726	6.7	431	8.5	1157	7.3
2016	1190	11.0	568	11.2	1758	11.1
2017	1107	10.2	543	10.7	1650	10.4
2018	1163	10.8	643	12.6	1806	11.4
2019	1003	9.3	514	10.1	1517	9.6
2020	942	8.7	466	9.2	1408	8.9
2021	1164	10.8	579	11.4	1743	11.0
2022	1099	10.2	473	9.3	1572	10.0
2023	958	8.9	443	8.7	1401	8.9
Total	10,813		5086		15,889	

*n* number of animals

Faecal consistency (Bristol Stool Chart): scale from 1 to 7; where 1–2 indicates very firm stools, 3–4 normal stools and 5–7 soft to liquid stools

Total may vary owing to missing data in some categories

**Table 3:** Global prevalence of intestinal parasites detected in canine and feline faecal samples analysed

		Dogs (*N* = 10,813)			Cats (*N* = 5086)			Total (*N* = 15,889)		
		*n*	%	95% CI	*n*	%	95% CI	*n*	%	95% CI
Protozoa	*G. duodenalis*	1732	16.0	15.3–16.7	332	6.5	5.8–7.2	2064	13.0	12.5–13.5
	*Cryptosporidium* spp.^*^	19	1.8	0.99–2.6	15	3.5	1.7–5.2	34	2.3	1.5–3.0
	*Cystoisospora* spp.	526	4.9	4.4–5.3	395	7.8	7.1–8.5	921	5.8	5.4–6.2
Nematoda	*Toxocara canis*/*cati*	296	2.7	2.3–3.0	388	7.6	6.9–8.3	684	4.3	4.0–4.6
	*Toxascaris leonina*	184	1.7	1.4–1.9	13	0.3	0.1–0.4	197	1.2	1.0–1.4
	Fam. Ancylostomatidae	267	2.5	2.2–2.7	23	0.5	0.3–0.7	290	1.8	1.6–2.0
	*Trichuris vulpis*	133	1.2	0.9–1.4	4	0.1	0.0–0.2	137	0.9	0.8–1.0
	*Eucoleus aerophilus*	8	0.1	0.0–0.2	8	0.2	0.1–0.3	16	0.1	0.0–0.1
	*Angiostrongylus vasorum*^♦^	7	3.3	0.9–5.7	ND	ND	ND	ND	ND	ND
	*Aelurostrongylus abstrusus*^♦^	ND	ND	ND	42	13.9	10.0–17.8	ND	ND	ND
	*Troglostongylus* spp.^♦^	ND	ND	ND	2	0.7	0.0–1.6	ND	ND	ND
	*Strongyloides stercoralis*^♦^	9	4.2	1.5–6.9	ND	ND	ND	ND	ND	ND
Cestoda	Fam. Taeniidae	19	0.2	0.1–0.3	16	0.3	0.2–0.5	35	0.2	0.1–0.3
	*Dipylidium caninum*	18	0.2	0.1–0.3	3	0.1	0.0–0.2	21	0.1	0.0–0.1
	*Joyeuxiella* spp.	ND	ND	ND	20	0.4	0.2–0.6	20	0.1	0.0–0.1

^*^number of samples analysed for *Cryptosporidium* spp. by DFA: dogs: 1046; cats: 428; total: 1474

*N*: total number of samples from dogs and cats

*n*: number of positive animals

^♦^number of samples analysed for lungworms and *S. stercoralis* by the Baermann–Wetzel method: dogs: 213; cats: 303; total: 516

*CI* confidence interval, *ND* not detected

The most prevalent parasites were *G. duodenalis* (16.0%; 1732/10,813)) infecting dogs and *Cystoisospora* spp. (7.8%; 395/5086) and *T. cati* (7.6%; 388/5086) infecting cats. The least prevalent parasites were *E. aerophilus* (0.1%; 8/10,813) in dogs and *D. caninum* (0.1%; 3/5086) in cats.

Of the 3891 samples testing positive in dogs and cats, 531 (13.6%) were co-infections with more than one parasite. The most common co-infections were *G. duodenalis*/*Cystoisospora* spp. (30.5%; 162/531) in dogs, followed by *Cystoisospora* spp./*T. cati* (13.4%; 71/531) and *G. duodenalis*/*T. cati* (13.2%; 70/531) in cats. No oocysts compatible with *T. gondii* or DNA were detected in the faecal samples from 108 cats analysed by qPCR.

### Associated epidemiological variables

Our statistical analysis results regarding associations between infection by each parasite and epidemiological variables are presented in Tables 4 and 5.

**Table 4:** Intestinal parasites detected in dogs by sex, age, origin, faecal consistency and season of the analysis

Variable		*G. duodenalis*	Cryptosporidium spp. % (+/*n*)^*^	*Cystoisospora* spp.	*T. canis*	*T. leonina*	Fam. Ancylostomatidae	*T. vulpis*	*E. aerophilus*	Fam. Taeniidae	*D. caninum*	*A. vasorum* % (+/*n*)^*^	*S. stercoralis* % (+/*n*)^*^
Age	< 1 (*n* = 489)	31.7 (155) ^a^	10 (6/60) ^a^	14.5 (71)^a^	5.5 (27) ^a^	1.4 (7)	2.2 (11)	0.8 (4)	0 (0)	0 (0)	0 (0)	0 (0/5)	40.0 (2/5) ^a^
	≥ 1 (*n* = 4162)	19.7 (819) ^b^	1.0 (7/685) ^b^	3.8 (157)^b^	1.4 (59) ^b^	0.9 (38)	1.9 (77)	0.5 (22)	0.02 (1)	0.3 (12)	0.02 (1)	0 (0/26)	0 (0/26) ^b^
	Total (*n* = 4651)	20.9 (974)	1.7 (13/745)	4.9 (228)	1.8 (86)	1.0 (45)	1.9 (88)	0.6 (26)	0.02 (1)	0.3 (12)	0.02 (1)	0 (0/31)	6.5 (2/31)
	*P*-value	** < 0.001**	** < 0.001**	** < 0.001**	** < 0.001**	0.26	0.53	0.41	0.1	0.23	0.73	–	** < 0.001**
Sex	Male (*n* = 4682)	15.8 (738)	2.9 (14/488) ^a^	4.8 (223)	3.1 (144)	1.9 (87)	2.2 (102)	1.3 (62)	0 (0)	0.2 (8)	0.3 (12)	3.8 (4/106)	1.9 (2/104)
	Female (*n* = 4806)	15.2 (729)	0.9 (4/458) ^b^	5.2 (249)	2.6 (125)	1.7 (83)	2.8 (133)	1.2 (57)	0.02 (1)	0.2 (8)	0.1 (5)	3.1 (3/97)	2.1 (2/95)
	Total (*n* = 9488)	15.5 (1467)	1.9 (18/946)	5.0 (472)	2.8 (269)	1.8 (170)	2.5 (235)	1.3 (119)	0.01 (1)	0.2 (16)	0.2 (17)	3.4 (7/203)	4.4 (9/203)
	*P*-value	0.42	**0.024**	0.3	0.16	0.63	0.065	0.54	0.45	0.96	0.08	0.79	0.92
Origin	Breeding (*n* = 2294)	22.5 (517) ^a^	0.2 (1/482) ^b^	4.7 (107)	0.7 (16) ^b^	0.3 (7) ^b^	1.2 (27)	0.1 (3) ^b^	0 (0)	0.1 (2)	0 (0) ^b^	0 (0/7)	0 (0/7)
	Owned (*n* = 2977)	15.8 (470)	3.3 (16/480) ^a^	5.3 (158)	2.9 (87)	1.0 (29) ^b^	1.6 (49)b	0.5 (16) ^b^	0.03 (1)	0.1 (3)	0.4 (11) ^a^	3.4 (7/203)	4.4 (9/203)
	Sheltered (*n* = 5399)	12.8 (693) ^b^	1.4 (1/74)	4.8 (258)	3.6 (192) ^a^	2.7 (148) ^a^	3.5 (189) ^a^	2.0 (110) ^a^	0.13 (7) ^a^	0.3 (14) ^a^	0.1 (7)	0 (0/3)	0 (0/3)
	Total (*n* = 10,670)	15.7 (1680)	1.7 (18/1036)	4.9 (523)	2.8 (295)	1.7 (184)	2.5 (265)	1.2 (129)	0.07 (8)	0.2 (19)	0.2 (18)	3.3 (7/213)	4.2 (9/213)
	*P*-value	** < 0.001**	** < 0.001**	0.5	** < 0.001**	** < 0.001**	** < 0.001**	** < 0.001**	0.1	0.13	**0.003**	0.84	0.8
Faecal consistency	1–2 (*n* = 61)	6.6 (4) ^b^	0.0 (0/)	0 (0)	0 (0)	0 (0)	0 (0)	0 (0)	0 (0)	0 (0)	0 (0)	0 (0/2)	0 (0/2)
	3–4 (*n* = 2829)	16.2 (458) ^b^	1.3 (8/632)	2.3 (66) ^b^	1.1 (31) ^b^	0.8 (23) ^b^	2.0 (57)	0.6 (17)	0.1 (2)	0.1 (4)	0.07 (2)	0 (0/58)	3.4 (2/58)
	5–7 (*n* = 1884)	22.6 (425) ^a^	2.7 (10/377)	6.9 (130) ^a^	2.9 (55) ^a^	1.8 (34) ^a^	2.7 (50)	0.7 (13)	0.1 (2)	0.1 (1)	0 (0)	3.8 (2/52)	3.8 (2/52)
	Total (*n* = 4774)	18.6 (887)	1.7 (18/)	4.1 (196)	1.8 (86)	1.2 (57)	2.2 (107)	0.6 (30)	0.1 (4)	0.1 (5)	0.04 (2)	1.8 (2/112)	3.6 (4/112)
	*P*-value	** < 0.001**	0.145	** < 0.001**	** < 0.001**	**0.006**	0.17	0.76	0.89	0.63	0.5	0.3	0.95
Season	Winter (*n* = 2674)	18.1 (483) ^a^	3.9 (12/310) ^a^	3.7 (100) ^b^	3.0 (79)	1.7 (46)	2.4 (63)	1.5 (39)	0.0 (1)	0.1 (2)	0.1 (3)	5.8 (3/52)	3.8 (2/52)
	Spring (*n* = 2969)	16.9 (501)	0.3 (1/363) ^b^	4.3 (129)	2.0 (59) ^b^	1.4 (41)	2.0 (59)	1.2 (35)	0.1 (3)	0.2 (7)	0.3 (10)	3.9 (2/51)	5.9 (3/51)
	Summer (*n* = 2091)	12.6 (263) ^b^	0.7 (1/135)	5.4 (112)	2.9 (61)	1.5 (32)	2.4 (50)	0.8 (16) ^b^	0.0 (0)	0.2 (5)	0.1 (3)	0 (0/56)	7.1 (4/56)
	Autumn (*n* = 3079)	15.8 (485)	2.1 (5/238)	6.0 (185) ^b^	3.2 (97)	2.1 (65) ^a^	3.1 (95) ^a^	1.4 (43)	0.1 (4)	0.2 (5)	0.1 (2)	3.7 (2/54)	0 (0/54) ^b^
	Total (*n* = 10,813)	11.6 (1249)	0.7 (7/1046)	3.9 (426)	2.0 (217)	1.3 (138)	1.9 (204)	0.9 (94)	0.1 (7)	0.2 (17)	0.1 (15)	1.9 (4/213)	3.3 (7/213)
	*P*-value	** < 0.001**	** < 0.001**	** < 0.001**	** < 0.001**	** < 0.001**	** < 0.001**	** < 0.001**	0.23	0.35	**0.02**	0.058	0.13

Bold indicates statistical significance

*n*: number of animals

+: number of positive animals

Faecal consistency: scale from 1 to 7, where 1–2 indicates very firm stools, 3–4 normal stools and 5–7 soft to liquid stools

^a^adjusted residual > 2: statistically significantly higher than expected

^b^adjusted residual ≤ 2: statistically significantly lower than expected

**Table 5:** Intestinal parasites detected in cats by sex, age, origin, faecal consistency and season of the analysis

Variable		*G. duodenalis*	*Cryptosporidium* spp. % (+/*n*)^*^	*Cystoisospora* spp.	*T. cati*	*T. leonina*	Fam. Ancylostomatidae	*T. vulpis*	*E. aerophilus*	Fam. Taeniidae	*D. caninum*	*Joyeuxiella* spp.	*A. abstrusus* % (+/*n*)^*^	*Troglostrongylus* spp. % (+/*n*)^*^
Age	< 1 (*n* = 276)	11.6 (32)	8.0 (2/25)	16.3 (45)^a^	11.2 (31)^a^	0.4 (1)	0 (0)	0 (0)	0.7 (2)^a^	0 (0)	0 (0)	0 (0)	25.0 (3/12)	0 (0/12)
	≥ 1 (*n* = 1053)	13.2 (139)	3.4 (11/321)	2.6 (27)^b^	4.4 (46)^b^	0.5 (5)	0.9 (9)	0.09 (1)	0 (0)^b^	0.3 (3)	0 (0)	0.2 (2)	7.6 (8/105)	0 (0/105)
	Total (*n* = 1329)	12.9 (171/)	3.8 (13/346)	5.4 (72)	5.8 (77)	0.5 (6)	0.7 (9)	0.08 (1)	0.2 (2)	0.2 (3)	0 (0)	0.2 (2)	9.4 (11/117)	0 (0/117)
	*P*-value	0.47	0.24	** < 0.001**	** < 0.001**	0.8	0.12	0.6	0.006	0.37	–	0.468	0.05	–
Sex	Male (*n* = 2223)	7.1 (158)	2.8 (6/212)	8.2 (182)	8.8 (196)^a^	0.2 (5)	0.3 (7)	0.04 (1)	0.22 (5)	0.1 (2)^b^	0.09 (2)	0.6 (14)	13.1 (19/145)	0 (0/145)
	Female (*n* = 2357)	6.2 (147)	4.9 (9/184)	7.6 (179)	7.1 (168)^b^	0.3 (8)	0.5 (12)	0.08 (2)	0.08 (2)	0.4 (10)^a^	0.04 (1)	0.3 (6)	13.1 (17/130)	1.5 (0/130)
	Total (*n* = 4580)	6.7 (305)	3.8 (15/396)	7.9 (3)	7.9 (364)	0.3 (13)	0.4 (19)	0.07 (3)	0.15 (7)	0.3 (12)	0.07 (3)	0.4 (20)	13.1 (36/275)	0.7 (2/275)
	*P*-value	0.23	0.28	0.45	**0.03**	0.46	0.306	0.59	0.22	0.03	0.53	0.054	0.99	0.133
Origin	Stray cats (*n* = 762)	12.7 (97)^a^	1.9 (4/214)	1.7 (13) ^b^	2.6 (20)^b^	0.7 (5)^a^	0.8 (6)	0 (0)	0 (0)	0.1 (1)	0 (0)	0.1 (1)	5.6 (5/90)^b^	0 (0/90)
	Owned (*n* = 1070)	6.1 (65)	3.9 (6/153)	5.3 (57)^b^	7.3 (78)	0.3 (3)	0.4 (4)	0.09 (1)	0.3 (3)	0.2 (2)	0 (0)	1.0 (11)^a^	17.7 (29/164)	0.6 (1/164)
	Sheltered (*n* = 3254)	5.2 (170)^b^	8.2 (5/41)^a^	10.0 (325) ^a^	8.9 (290)^a^	0.2 (5)	0.4 (13)	0.09 (1)	0.2 (5)	0.4 (13)	0. 09 (3)	0.3 (9)^b^	16.3 (8/49)^a^	2.0 (1/49)
	Total (*n* = 5086)	6.5 (332)	3.5 (15/428)	7.8 (395)	7.6 (388)	0.3 (13)	0.5 (23)	0.08 (4)	0.2 (8)	0.3 (16)	0.06 (3)	0.4 (21)	13.9 (42/303)	0.7 (2/303)
	*P*-value	** < 0.001**	0.056	** < 0.001**	** < 0.001**	**0.046**	0.324	0.7	0.32	0.35	0.43	**0.0016**	**0.024**	0.36
Faecal consistency	1–2 (*n* = 194)	3.1 (6)	12.5 (1/8)	9.8 (19) ^a^	9.3 (18)^a^	0 (0)	0.5 (1)	0 (0)	0.5 (1)	0 (0)	0 (0)	1.5 (3)	23.5 (4/17)	0 (0/17)
	3–4 (*n* = 1414)	5.7 (81)	3.1 (1/32)	4.1 (58)	5.5 (78)	0.3 (4)	0.4 (6)	0.07 (1)	0.1 (2)	0.1 (2)	0 (0)	0.7 (10)	10.7 (15/140)	0 (0/140)
	5–7 (*n* = 329)	13.7 (45)^a^	0 (0/4)	2.7 (9)	5.5 (18)	0.3 (1)	0.3 (1)	0 (0)	0.6 (2)	0.3 (1)	0 (0)	1.2 (4)	21.1 (4/19)	5.3 (1/19)
	Total (*n* = 1937)	6.8 (132)	4.5 (2/44)	4.4 (86)	5.9 (114)	0.3 (5)	0.4 (8)	0.05 (1)	0.3 (5)	0.2 (3)	0 (0)	0.9 (17)	13.1 (23/176)	0.6 (1/176)
	*P*-value	** < 0.001**	0.47	** < 0.001**	0.106	0.75	0.928	0.83	0.24	0.67	–	0.386	0.184	**0.015**
Season	Winter (*n* = 1095)	10.4 (114)^a^	2.8 (5/179)	4.3 (47)^b^	5.5 (60)^b^	0.8 (9)^b^	0.6 (7)	0.2 (2)	0.2 (2)	0 (0)^b^	0.2 (2)	0.5 (5)	23.9 (11/46)	0 (0/46)
	Spring (*n* = 1157)	4.0 (46)	1.2 (1/83)	8.8 (102)	7.0 (81)	0.1 (1)	0.4 (5)	0 (0)	0.2 (2)	0.2 (2)	0 (0)	0.3 (4)	15.4 (12/78)	1.3 (1/78)
	Summer (*n* = 1438)	5.6 (81)^b^	2.7 (1/37)	8.9 (128)	8.7 (125)	0.1 (2)	0.2 (3)	0.1 (1)	0.2 (3)	0.3 (4)	0.1 (1)	0.1 (2)	17.2 (10/58)	1.7 (1/58)
	Autumn (*n* = 1396)	6.5 (91)	6.2 (8/129)	8.5 (118)	8.7 (122)	0.1 (1)	0.6 (8)	0.1 (1)	0.1 (1)	0.7 (10)^a^	0 (0)	0.7 (10)^a^	7.4 (9/121)	0 (0/121)
	Total (*n* = 5086)	4.3 (218)	2.3 (10/428)	6.8 (348)	6.4 (328)	0.1 (4)	0.3 (16)	0 (1)	0.1 (6)	0.3 (16)	0 (1)	0.3 (16)	10.2 (31/303)	0.7 (2/303)
	*P*-value	** < 0.001**	**0.03**	** < 0.001**	** < 0.001**	** < 0.001**	0.053	0.07	0.6	0.009	** < 0.001**	**0.025**	**0.001**	0.46

Bold indicates statistical significance

^*^the total number of samples analysed for *Cryptosporidium* spp. analyses differs from that for the other parasites. The number of animals analysed is indicated as *n* in the format (+/*n*) for each variable

+: number of positive animals

*n*: number of animals

Faecal consistency: scale from 1 to 7, where 1–2 indicates very firm stools, 3–4 normal stools and 5–7 soft to liquid stools

^a^adjusted residual > 2: statistically significantly higher than expected

^b^adjusted residual ≤ 2: statistically significantly lower than expected

### Dogs

Young dogs (< 1 year) showed a significantly higher risk of infection by *Cystoisospora* spp. (*χ*^2^ = 108,19; *df * = 1; *P* = < 0.001), *G. duodenalis* (*χ*^2^ = 37.96; *df* = 1; *P* = < 0.001), *Cryptosporidium* spp. (*χ*^2^ = 25.78; *df* = 1; *P* = < 0.001), *T. canis* (*χ*^2^ = 40.35; *df* = 1; *P* = < 0.001) and *S. stercoralis* (*χ*^2^ = 11.19; *df* = 1; *P* = < 0.001). The risk of *G. duodenalis* infection was higher in dogs from breeding (*χ*^2^ = 114.07; *df* = 2; *P* = < 0.001), while *Cryptosporidium* spp. (*χ*^2^ = 13.83; *df* = 2; *P* = < 0.001) and *D. caninum* (*χ*^2^ = 11.61; *df* = 2; *P* = 0.003) were more frequently detected in owned dogs. Among shelter dogs, a higher positivity rate was recorded for *T. canis* (*χ*^2^ = 48.90; *df* = 2; *P* = < 0.001), *T. leonina* (*χ*^2^ = 69.93; *df* = 2; *P* = < 0.001), ancylostomids (*χ*^2^ = 49.07; *df* = 2; *P* = < 0.001) and *T. vulpis* (*χ*^2^ = 64.60; *df* = 2; *P* = < 0.001). *Cystoisospora* spp. (*χ*^2^ = 60.25; *df *= 2; *P* = < 0.001), *G. duodenalis* (*χ*^2^ = 36.91; *df* = 2; *P* = < 0.001), *T. canis* (*χ*^2^ = 22.95; *df* = 2; *P* = < 0.001) and *T. leonina* (*χ*^2^ = 10.27; *df* = 2; *P* = 0.006) were more frequently detected in samples from dogs with soft stools (consistency scores of 5–7) than normal stools (scores of 1–4). Regarding seasonality, *G. duodenalis* was more prevalent in the winter (*χ*^2^ = 238.80; *df* = 3; *P* = < 0.001), whereas *Cystoisospora* (*χ*^2^ = 47.95; *df* = 3; *P* < 0.001), *T. leonina* (*χ*^2^ = 22.34; *df* = 3; *P* = < 0.001) and ancylostomids (*χ*^2^ = 29.24; *df* = 3; *P* = < 0.001) were more prevalent in the autumn.

### Cats

A significantly higher-than-expected prevalence of *T. cati* infection was observed in male cats (*χ*^2^ = 4.48; *df* = 1; *P* = 0.03). Kittens (< 1 year) had a higher risk of infection by *Cystoisospora* spp. (*χ*^2^ = 80.90; *df* = 1; *P* = < 0.001) and *T. cati* (*χ*^2^ = 18.99; *df *= 1; *P* < 0.001). Cats from feline colonies were found to be more frequently infected by *G. duodenalis* (*χ*^2^ = 57.60; *df* = 2; *P* < 0.001) and *T. leonina* (*χ*^2^ = 6.16; *df* = 2; *P* = 0.046), while *Cystoisospora* spp. (*χ*^2^ = 67.42; *df* = 2; *P* = < 0.001) and *T. cati* (*χ*^2^ = 35.47; *df* = 2; *P* = < 0.001) infections were more prevalent in shelter cats. In cats with soft stools (score 5–7), infection by *G. duodenalis* was more prevalent (*χ*^2^ = 31.15; *df* = 2; *P* < 0.001), whereas in cats with hard dry stools (score 1–2), infections by *Cystoisospora* spp. (*χ*^2^ = 15.65; *df* = 2; *P* = < 0.001) were more prevalent. As in the case of dogs, the prevalence of *G. duodenalis* infection was higher in winter (*χ*^2^ = 122.04; *df* = 3; *P* = < 0.001) and that of *Joyeuxiella* spp. (*χ*^2^ = 9.36; *df* = 3; *P* = 0.025) was higher in autumn.

### Trends in parasite infection prevalences over the years

**Fig. 3:**
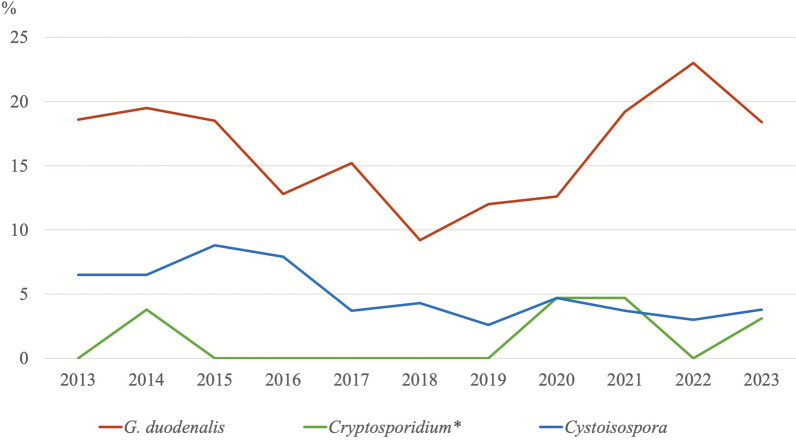
Trends in the percentages of protozoan parasites detected in dogs over time. ^*^The number of samples analysed for the diagnosis of *Cryptosporidium* spp. is not the same as for the other parasites. Details are presented in Table 3

**Fig. 4:**
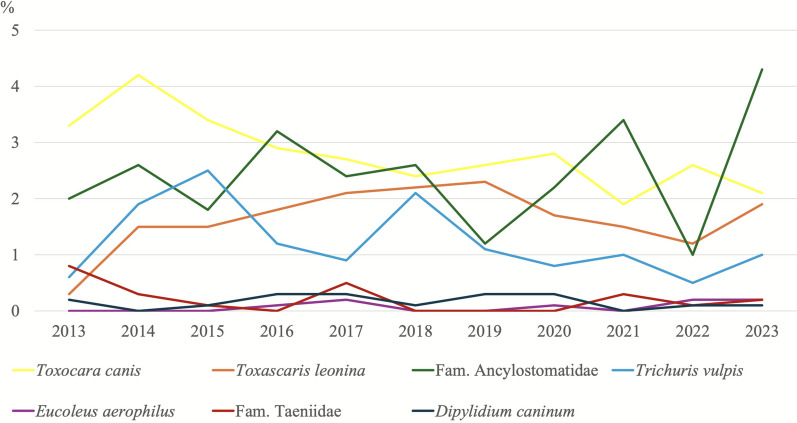
Trends in the percentages of helminth parasites detected in dogs over time

**Fig. 5:**
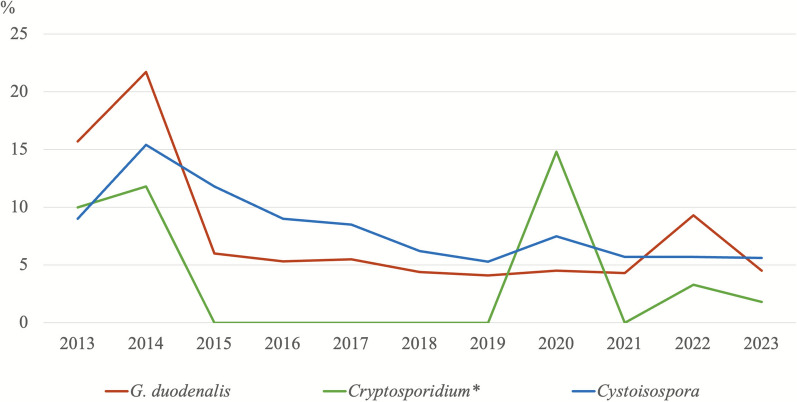
Trends in the percentages of protozoan parasites detected in cats over time. ^*^The number of samples analysed for the diagnosis of *Cryptosporidium* spp. is not the same as for the other parasites. Details are presented in Table 3

**Fig. 6:**
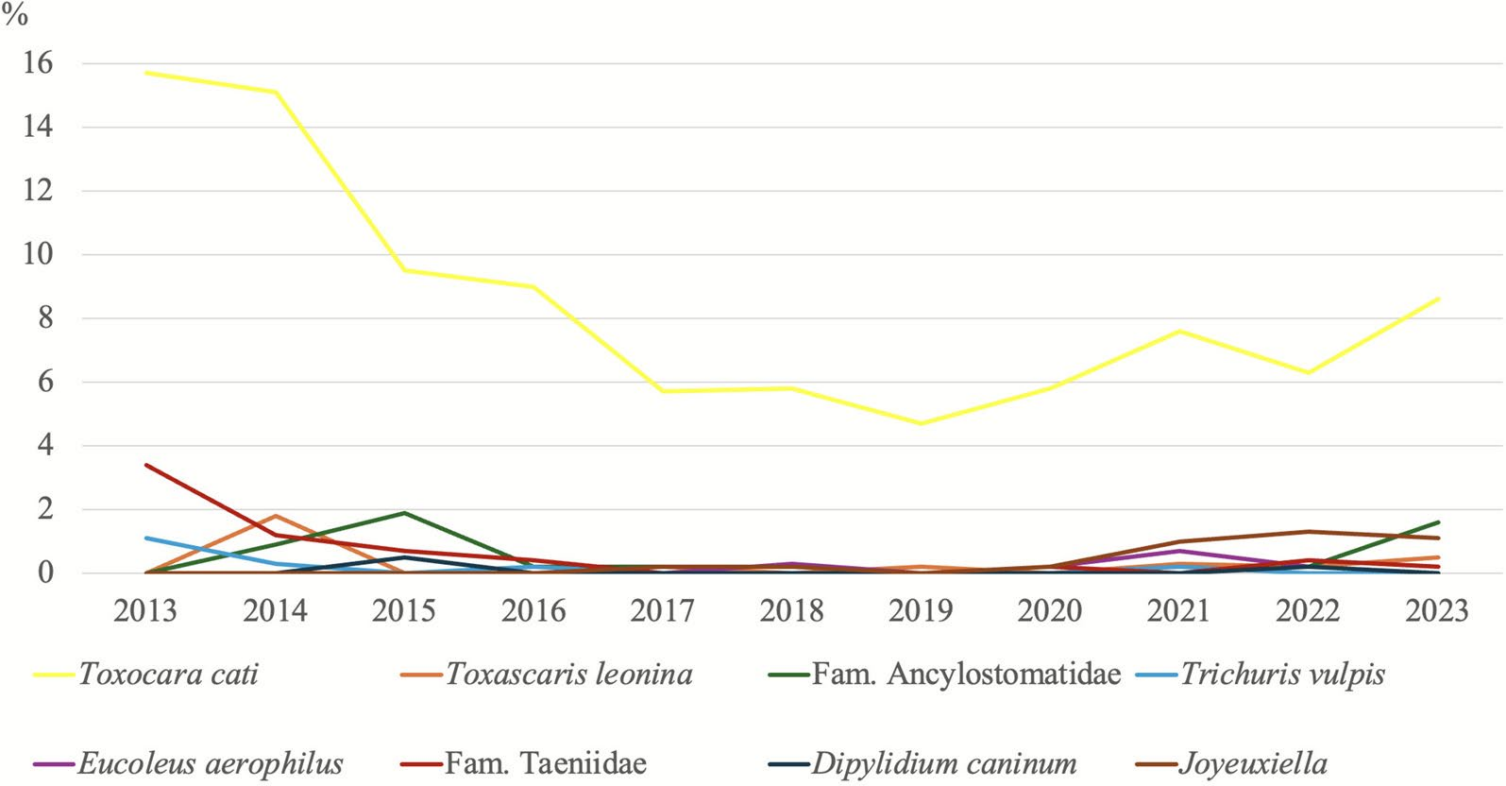
Trends in the percentages of helminth parasites detected in cats over time

Figures 3 and 4 show the prevalence data of protozoan and helminth infections detected in canine samples throughout the study period, while Figs. 5 and 6 present the corresponding prevalence data obtained from feline samples.

In dogs, the prevalence of *G. duodenalis* infection was higher than expected in 2021, 2022 and 2023 (*χ*^2^ = 137.08; *df* = 10; *P* = < 0.001), whereas the prevalence of *Cystoisospora* spp. showed a decreasing trend in 2021 and 2022 (*χ*^2^ = 86.14; *df* = 10; *P* = < 0.001). In the case of helminth infections, only those caused by ancylostomids showed a higher-than-expected prevalence in 2023 in both dogs (*χ*^2^ = 39.25; *df *= 10; *P* = < 0.001) and cats (*χ*^2^ = 42.47; *df* = 10; *P* = < 0.001).

*Joyeuxiella* infection in cats showed a clear increasing trend in the recent years (from 2020 to the present).

## Discussion

This is the largest epidemiological survey of endoparasites in dogs and cats based on coprological data conducted in Spain. Our results provide valuable insight into the prevalence and epidemiological trends of intestinal parasites in dogs and cats living in Madrid over a 10-year period. The significant variations in parasite prevalences, co-infections and associated epidemiological factors identified here contribute to a better understanding of intestinal parasite infections in companion animals.

The overall prevalence of parasites in dogs (26%) was higher than in cats (21.4%), consistent with findings from other large-scale studies [30–32]. These differences may be attributed to factors such as study area, diagnostic techniques and population characteristics (e.g. age and housing conditions) [33]. However, it is important to consider the limitations of coprological methods, particularly their sensitivity for detecting parasites with intermittent shedding patterns which may lead to underdiagnoses [34].

In dogs, the most prevalent parasite detected was *G. duodenalis* (16.0%), whereas in cats, *Cystoisospora* spp. (7.8%) and *T. cati* (7.6%) were the most frequently detected. These findings are in line with data from previous studies indicating that *G. duodenalis* is a widespread protozoan parasite in dogs, particularly in environments with high animal densities such as shelters and breeding facilities [35]. Similarly, *Cystoisospora* spp. is known to be a prevalent parasite in feline populations, especially among kittens, cats with gastrointestinal disorders and shelter animals [32, 36, 37]. The lower detection rate of helminth parasites may be related to deworming protocols. These are routinely used in pets but do not typically include chemicals that prevent infection by protozoa. Veterinarians must therefore interpret the results of a coprological analysis and decide whether treatment against protozoal infections is necessary in otherwise healthy animals. Emphasis should be placed on preventing protozoan infections through other strategies such as proper hygiene and faecal removal from the environment [38].

Interestingly, the less prevalent parasites detected in dogs and cats were *E. aerophilus* (0.1% and 0.2%, respectively), in agreement with reports of studies in dogs samples [39, 40]. In cats, the least prevalent parasite was *D. caninum* (0.1%). This suggests that these parasites have low transmission rates in this species. In the case of *D. caninum* in cats, this could be related to effective flea control measures. Alternatively, *D. caninum* prevalence may be underestimated because of detection difficulties arising from the intermittent shedding of proglottids in faeces and the rupture of the ovigerous capsule, as the eggs may be indistinguishable from those of other cestode species [41].

The most common co-infections observed in dogs were *G. duodenalis*/*T. canis*, while in cats, they were *Cystoisospora* spp./*T. cati* and *Cystoisospora* spp./*G. duodenalis.* These results highlight *G. duodenalis* as one of the most prevalent parasites in companion animals in the last decade, with significance implications not only in mono-infections but also in co-infections with other intestinal parasites. Controlling this parasite is crucial, as a positive result for another parasite could mask the presence of this potentially zoonotic pathogen, posing public health risks, especially in households where dogs and cats live with people at risk (e.g. children, pregnant women, immunocompromised individuals or elderly people). Our data indicate that specific parasites co-exist in the same hosts, likely owing to similar transmission routes or overlapping environmental conditions. The relatively low prevalence of co-infections, however, suggests that mono-infections were common in our study sample, in line with the findings of previous studies in the same area [42].

Prevalence data for lung worms detected by the Baermann–Wetzel method should be interpreted with caution, as the total sample size differs from that of other analyses. This is because the Baermann–Wetzel method is not a routine procedure and was only requested when there was specific suspicion of infection. In addition, the absence of *T. gondii* in all feline faecal samples should be underscored although expected, given the results of other authors [43]. This could be owing to the limited shedding period of *T. gondii* oocysts in cats or a low environmental contamination level during the sampling period. Further work is needed with a larger sample size and different diagnostic methods for a more comprehensive analysis of *T. gondii* prevalence in cats and the importance of its transmission to humans [44].

The analysis of the epidemiological variables studied served to identify significant correlations between host characteristics and the parasite detected. Age played a key role in infection risk, such that puppies and kittens under 10 year of age were more susceptible to infections, such as *Cystoisospora* spp., *G. duodenalis*, *Cryptosporidium* spp., *T. canis* and *S. stercoralis* infection in dogs and *Cystoisospora* spp. and *T. cati* infection in cats. This higher susceptibility is most likely due to an immature immune system and increased exposure to parasites in crowded environments [45, 46]. We should also consider the role of transplacental transmission in *T. canis* and lactogenic transmission in both *T. canis* and *T. cati*, as these are critical transmission routes in young animals [11].

Housing conditions also influenced infection risk. Dogs from breeders had a higher prevalence of *G. duodenalis* infection, but in this case, owned animals showed higher rates than those from the shelter. This could be because owned puppies were often brought in for deworming prior to vaccination and frequently originated from breeding facilities where prevalence is higher. In contrast, the shelter submitting samples implemented very strict control measures, both for the animals and the environment, which likely limited parasite transmission. Shelter dogs were more frequently infected by *T. canis*, *T. leonina*, ancylostomids, *T. vulpis* and *E. aerophilus*. In contrast, owned dogs showed a higher prevalence of *Cryptosporidium* spp. and *D. caninum* infection, most likely because of ease of direct transmission due to closer contact between animals and even humans. As for *D. caninum* infection, improved household conditions help maintain the life cycle of fleas, facilitating the persistence of eggs, larval and pupal stages of the fleas in the environment and, although most pet owners deworm their pets, they do so on the basis of very different criteria and often irregularly [47, 48]. Similarly, cats from colonies featured higher infection rates of *G. duodenalis* and *T. leonina*, whereas shelter cats had a greater prevalence of *Cystoisospora* spp. and *T. cati*. This underscores the impact of environmental factors, population density and the lack of awareness of pet owners of parasite transmission [49] and highlights the importance of parasite control in pets owing to zoonotic risks [50, 51].

Faecal consistency (according to the Bristol Stool Chart) was also found to correlate with infection prevalence. Hence, dogs with soft stools (scores 5–7) had higher detection rates of *Cystoisospora* spp., *G. duodenalis*, *T. canis* and *T. leonina*, while in cats, *G. duodenalis* was more common in soft stools and *Cystoisospora* spp. and *T. cati* were more frequent in hard dry faeces samples (scores 1–2). This suggests that certain parasites contribute to gastrointestinal disturbances, while others may cause subclinical disease [52]. Accordingly, we would recommend routine coprological tests on ‘clinically healthy’ animals with faeces with normal characteristics, especially in cats, as they usually defecate in litter boxes and depending on the composition of the litter the organoleptic properties of the faeces may be modified making their consistency classification more difficult.

Seasonality in prevalence was observed, *G. duodenalis* infection being more prevalent in winter and *T. leonina* and ancylostomids in autumn in both dogs and cats. The higher winter prevalence of *G. duodenalis* may be explained by increased indoor housing, leading to higher transmission rates, whereas autumn peaks in helminths could be related to increased environmental contamination in this season because the climate conditions are more favourable for the survival of infective larval stages [53].

Longitudinal analysis of data collected from 2013 to 2023 revealed fluctuations in parasite prevalences. In dogs, *G. duodenalis* showed a higher-than-expected increase in 2021–2023, suggesting a rising trend, possibly attributable to increased awareness and improved diagnostic techniques compared with early years [54]. Conversely, *Cystoisospora* spp. in dogs showed a decreasing trend in 2021–2022. This may be attributed to improved hygiene practices and management strategies in shelter animals [55]. Helminth infections, particularly ancylostomids infections, experienced a significant increase in 2023 in both dogs and cats. This could suggest their re-emergence, possibly due to environmental or climate factors [56]. Notably, *Joyeuxiella* spp. infection in cats showed a consistent increase from 2020 to the present, warranting further investigation into their epidemiology and potential risk factors for transmission.

## Conclusions

This study provides updates and comprehensive data regarding the prevalence of endoparasites and their co-infections and epidemiological trends in dogs and cats over a decade. *G. duodenalis* emerged as the most prevalent parasite detected in both dogs and cats living in Madrid. These findings highlight the importance of regular parasitological surveillance, targeted deworming protocols and improved hygiene measures to control parasite transmission and reduce associated zoonotic risks.

The increasing trend in pet ownership highlights the need for increased awareness and preventive strategies to address the public health challenges posed by parasite infections in companion animals. Future research should focus on identifying emerging trends, assessing the impact of climate change on parasite epidemiology and exploring host–parasite interactions to enhance prevention and control strategies for companion animals. By adopting a One Health approach that collectively considers animal, human and environmental health, we can protect both companion animals and their owners, reducing the impacts of parasite diseases on public health.

These findings highlight the importance of regular coprological analysis for the detection of intestinal parasites, either in veterinary practices or via reference laboratories. Such monitoring is valuable for quality control of established deworming protocols, allowing confirmation of their effectiveness or the need for protocol adjustments.

## Data Availability

Data supporting the main conclusions of this study are included in the manuscript.

## Abbreviations

COVID: Coronavirus disease
MIF: Merthiolate–iodine–formalin
PCR: Polymerase chain reaction
DFA: Direct fluorescent assay
CI: Confidence interval
NCBI: National Center for Biotechnology Information
BLAST: Basic Local Alignment Search Tool
SPSS: Statistical Package for the Social Sciences
ESCCAP: European Scientific Counsel Companion Animal Parasites

## References

[1] Ministerio de Derechos Sociales, Consumo y Agenda 2030-ANFAAC. https://www.mdsocialesa2030.gob.es/index.htm. Accessed 4 Dec 2024.

[2] Jones KE, Patel NG, Levy MA, Storeygard A, Balk D, Gittleman JL, et al. Global trends in emerging infectious diseases. Nature. 2008;451:990–3. 18288193 10.1038/nature06536 PMC5960580

[3] Papaiakovou M, Pilotte N, Grant JR, Traub RJ, Llewellyn S, McCarthy JS, et al. A novel, species-specific, real-time PCR assay for the detection of the emerging zoonotic parasite *Ancylostoma ceylanicum* in human stool. PLoS Negl Trop Dis. 2017;11:e0005734. 10.1371/journal.pntd.0005734. 28692668 10.1371/journal.pntd.0005734 PMC5519186

[4] American Animal Hospital Association-American Veterinary Medical Association Preventive Healthcare Guidelines Task Force. Development of new canine and feline preventive healthcare guidelines designed to improve pet health. J Am Anim Hosp Assoc. 2011;47:306–11. 10.5326/JAAHA-MS-4007 21896837

[5] Miró G, Gálvez R, Montoya A, Delgado B, Drake J. Survey of Spanish pet owners about endoparasite infection risk and deworming frequencies. Parasit Vectors. 2020;13:101. 32102683 10.1186/s13071-020-3976-8 PMC7045513

[6] Baneth G, Thamsborg SM, Otranto D, Guillot J, Blaga R, Deplazes P, et al. Major parasitic zoonoses associated with dogs and cats in Europe. J Comp Pathol. 2016;155:54–74. 10.1016/j.jcpa.2015.10.179 26687277

[7] Dixon BR. *Giardia duodenalis* in humans and animals-transmission and disease. Res Vet Sci. 2021;135:283–9. 10.1016/j.rvsc.2020.09.034. 33066992 10.1016/j.rvsc.2020.09.034

[8] Robertson ID, Irwin PJ, Lymbery AJ, Thompson RC. The role of companion animals in the emergence of parasitic zoonoses. Int J Parasitol. 2000;30:1369–77. 10.1016/s0020-7519(00)00134-x. 11113262 10.1016/s0020-7519(00)00134-x

[9] Sini MF, Tamponi C, Mehmood N, Dessì G, Ariu F, Carta C, et al. Laboratory associated zoonotic parasitic infections: a review of main agents and biosecurity measures. J Infect Dev Ctries. 2023;17:762–81. 37406067 10.3855/jidc.9428

[10] Dryden MW, Payne PA, Smith V. Accurate diagnosis of *Giardia* spp. and proper fecal examination procedures. Vet Ther. 2006;7:4–14. 16598679

[11] Deplazes P, Eckert J, Mathis A, von Samson-Himmelstjerna G, Zahner H. Parasitology in Veterinary Medicine. Wageningen: Wageningen Academic Publishers; 2016.

[12] Sykes JE. Greene’s Infectious Diseases of the Dog and Cat. Elsevier Health Sciences; 2022.

[13] Overgaauw PAM, Nederland V. Aspects of *Toxocara* epidemiology: toxocarosis in dogs and cats. Crit Rev Microbiol. 1997;23:233–51. 10.3109/10408419709115138. 9347222 10.3109/10408419709115138

[14] Macpherson CNL. The epidemiology and public health importance of toxocariasis: a zoonosis of global importance. Int J Parasitol. 2013;43:999–1008. 10.1016/j.ijpara.2013.07.004. 23954435 10.1016/j.ijpara.2013.07.004

[15] Drake J, Sweet S, Baxendale K, Hegarty E, Horr S, Friis H, et al. Detection of *Giardia* and helminths in Western Europe at local K9 (canine) sites (DOGWALKS Study). Parasit Vectors. 2022;15:311. 36057606 10.1186/s13071-022-05440-2 PMC9440314

[16] Lewis SJ, Heaton KW. Stool form scale as a useful guide to intestinal transit time. Scand J Gastroenterol. 1997;32:920–4. 10.3109/00365529709011203. 9299672 10.3109/00365529709011203

[17] McGrath K, Caldwell P. Diagnostic approach to constipation in children. In: Di Palma J, editor. *Constipation–Causes, Diagnosis and Treatment.* London: IntechOpen; 2012. 10.5772/29180.

[18] Cringoli G, Maurelli MP, Levecke B, Bosco A, Vercruysse J, Utzinger J, et al. The mini-FLOTAC technique for the diagnosis of helminth and protozoan infections in humans and animals. Nat Protoc. 2017;12:1723–32. 28771238 10.1038/nprot.2017.067

[19] Maurelli MP, Rinaldi L, Alfano S, Pepe P, Coles GC, Cringoli G. Mini-FLOTAC, a new tool for copromicroscopic diagnosis of common intestinal nematodes in dogs. Parasit Vectors. 2014;7:356. 25095701 10.1186/1756-3305-7-356 PMC4262189

[20] Thienpont D, Rochette F, Vanparijs OFJ. Diagnosing Helminthiasis through Coprological Examination. 1st ed. Beerse, Belgium: Janssen Research Foundation; 1978.

[21] Silva KJS, Sabogal-Paz LP. Analytical challenges and perspectives of assessing viability of *Giardia muris* cysts and *Cryptosporidium parvum* oocysts by live/dead simultaneous staining. Environ Technol. 2022;43:60–9. 10.1080/09593330.2020.1775712. 32463712 10.1080/09593330.2020.1775712

[22] Conboy GA. Canine angiostrongylosis: the French heartworm: an emerging threat in North America. Vet Parasitol. 2011;176:382–9. 10.1016/j.vetpar.2011.01.025. 21310537 10.1016/j.vetpar.2011.01.025

[23] Traversa D, Lepri E, Veronesi F, Paoletti B, Simonato G, Diaferia M, et al. Metastrongyloid infection by *Aelurostrongylus abstrusus*, *Troglostrongylus brevior* and *Angiostrongylus chabaudi* in a domestic cat. Int J Parasitol. 2015;45:685–90. 10.1016/j.ijpara.2015.05.005. 26149643 10.1016/j.ijpara.2015.05.005

[24] Verweij JJ, Schinkel J, Laeijendecker D, van Rooyen MAA, van Lieshout L, Polderman AM. Real-time PCR for the detection of *Giardia lamblia*. Mol Cell Probes. 2003;17:223–5. 14580396 10.1016/s0890-8508(03)00057-4

[25] Tiangtip R, Jongwutiwes S. Molecular analysis of *Cryptosporidium* species isolated from HIV-infected patients in Thailand. Trop Med Int Health. 2002;7:357–64. 11952952 10.1046/j.1365-3156.2002.00855.x

[26] Costa JM, Pautas C, Ernault P, Foulet F, Cordonnier C, Bretagne S. Real-time PCR for diagnosis and follow-up of *Toxoplasma* reactivation after allogeneic stem cell transplantation using fluorescence resonance energy transfer hybridization probes. J Clin Microbiol. 2000;38:2929–32. 10921953 10.1128/jcm.38.8.2929-2932.2000 PMC87150

[27] Verweij JJ, Canales M, Polman K, Ziem J, Brienen EAT, Polderman AM, et al. Molecular diagnosis of *Strongyloides stercoralis* in faecal samples using real-time PCR. Trans R Soc Trop Med Hyg. 2009;103:342–6. 19195671 10.1016/j.trstmh.2008.12.001

[28] Jothikumar N, Murphy JL, Hill VR. Detection and identification of *Giardia* species using real-time PCR and sequencing. J Microbiol Methods. 2021;189:106279. 10.1016/j.mimet.2021.106279. 34271057 10.1016/j.mimet.2021.106279

[29] Tamura K, Stecher G, Peterson D, Filipski A, Kumar S. MEGA6: molecular evolutionary genetics analysis version 6.0. Mol Biol Evol. 2013;30:2725–9. 24132122 10.1093/molbev/mst197 PMC3840312

[30] Barutzki D, Schaper R. Results of parasitological examinations of faecal samples from cats and dogs in Germany between 2003 and 2010. Parasitol Res. 2011;109:45–60. 10.1007/s00436-011-2402-8 21739375

[31] Lorenzini G, Tasca T, De Carli GA. Prevalence of intestinal parasites in dogs and cats under veterinary care in Porto Alegre, Rio Grande do Sul, Brazil. Braz J Vet Res Anim Sci. 2007;44:137–45.

[32] Villeneuve A, Polley L, Jenkins E, Schurer J, Gilleard J, Kutz S, et al. Parasite prevalence in fecal samples from shelter dogs and cats across the Canadian provinces. Parasit Vectors. 2015;8:281. 26013283 10.1186/s13071-015-0870-x PMC4451884

[33] Rodriguez JY, Cummings KJ, Hodo CL, Hamer SA. A repeated cross-sectional study of intestinal parasites in Texas shelter dogs using fecal flotation and saline sedimentation. Parasitol Res. 2023;122:237–43. 36372803 10.1007/s00436-022-07722-1 PMC9853879

[34] Palmer CS, Thompson RCA, Traub RJ, Rees R, Robertson ID. National study of the gastrointestinal parasites of dogs and cats in Australia. Vet Parasitol. 2008;151:181–90. 10.1016/j.vetpar.2007.10.015. 18055119 10.1016/j.vetpar.2007.10.015

[35] Bouzid M, Halai K, Jeffreys D, Hunter PR. The prevalence of *Giardia* infection in dogs and cats, a systematic review and meta-analysis of prevalence studies from stool samples. Vet Parasitol. 2015;207:181–202. 10.1016/j.vetpar.2014.12.011. 25583357 10.1016/j.vetpar.2014.12.011

[36] Genchi M, Vismarra A, Zanet S, Morelli S, Galuppi R, Cringoli G, et al. Prevalence and risk factors associated with cat parasites in Italy: a multicenter study. Parasit Vectors. 2021;14:475. 34526126 10.1186/s13071-021-04981-2 PMC8441231

[37] Ursache AL, Györke A, Mircean V, Dumitrache MO, Codea AR, Cozma V. *Toxocara cati* and other parasitic enteropathogens: more commonly found in owned cats with gastrointestinal signs than in clinically healthy ones. Pathogens. 2021;10:198. 33668439 10.3390/pathogens10020198 PMC7917965

[38] ESCCAP UK. http://www.esccapuk.org.uk. Accessed on 2025 Jun 10.

[39] Samorek-Pieróg M, Cencek T, Łabuć E, Pac-Sosińska M, Pieróg M, Korpysa-Dzirba W, et al. Occurrence of *Eucoleus aerophilus* in wild and domestic animals: a systematic review and meta-analysis. Parasit Vectors. 2023;16:245. 37475031 10.1186/s13071-023-05830-0 PMC10360280

[40] Simonato G, Cassini R, Morelli S, Di Cesare A, La Torre F, Marcer F, et al. Contamination of Italian parks with canine helminth eggs and health risk perception of the public. Prev Vet Med. 2019;172:104788. 10.1016/j.prevetmed.2019.104788. 31627164 10.1016/j.prevetmed.2019.104788

[41] Rousseau J, Castro A, Novo T, Maia C. *Dipylidium caninum* in the twenty-first century: epidemiological studies and reported cases in companion animals and humans. Parasit Vectors. 2022;15:131. 35534908 10.1186/s13071-022-05243-5 PMC9088078

[42] Mateo M, Montoya A, Bailo B, Köster PC, Dashti A, Hernández-Castro C, et al. Prevalence and public health relevance of enteric parasites in domestic dogs and cats in the region of Madrid (Spain) with an emphasis on *Giardia duodenalis* and *Cryptosporidium* sp. Vet Med Sci. 2023;9:2542–58. 10.1002/vms3.1270. 37725371 10.1002/vms3.1270 PMC10650246

[43] Zhu S, Shapiro K, VanWormer E. Dynamics and epidemiology of *Toxoplasma gondii* oocyst shedding in domestic and wild felids. Transbound Emerg Dis. 2022;69:2412–23. 10.1111/tbed.14197. 34153160 10.1111/tbed.14197

[44] Dabritz HA, Conrad PA. Cats and *Toxoplasma*: implications for public health. Zoonoses Public Health. 2010;57:34–52. 19744306 10.1111/j.1863-2378.2009.01273.x

[45] Barutzki D, Schaper R. Age-dependant prevalence of endoparasites in young dogs and cats up to one year of age. Parasitol Res. 2013;112:119–31. 23779224 10.1007/s00436-013-3286-6

[46] Gates MC, Nolan TJ. Endoparasite prevalence and recurrence across different age groups of dogs and cats. Vet Parasitol. 2009;166:153–8. 19709815 10.1016/j.vetpar.2009.07.041 PMC2783654

[47] Matos M, Alho AM, Owen SP, Nunes T, de Maira Carvalho L. Parasite control practices and public perception of parasitic diseases: a survey of dog and cat owners. Prev Vet Med. 2015;122:174–80. 26404913 10.1016/j.prevetmed.2015.09.006

[48] McNamara J, Drake J, Wiseman S, Wright I. Survey of European pet owners quantifying endoparasitic infection risk and implications for deworming recommendations. Parasit Vectors. 2018;11:571. 30382932 10.1186/s13071-018-3149-1 PMC6211546

[49] Zanzani SA, Gazzonis AL, Scarpa P, Berrilli F, Manfredi MT. Intestinal parasites of owned dogs and cats from metropolitan and micropolitan areas: prevalence, zoonotic risks, and pet owner awareness in Northern Italy. BioMed Res Int. 2014;2014:1–10. 10.1155/2014/696508. 10.1155/2014/696508 PMC4022196 24883320

[50] European Scientific Counsel Companion Animal Parasites (ESCCAP). Guideline 01: Worm Control in Dogs and Cats. 7th ed. Malvern, UK: ESCCAP; 2025. ISBN 978-1-913757-67-0.

[51] European Scientific Counsel Companion Animal Parasites (ESCCAP). Guideline 06: Control of Intestinal Protozoa in Dogs and Cats. 7th ed. Malvern, UK: ESCCAP; 2025. ISBN 978-1-913757-63-2.

[52] Tysnes KR, Skancke E, Robertson LJ. Subclinical *Giardia* in dogs: a veterinary conundrum relevant to human infection. Trends Parasitol. 2014;30:520–7. 10.1016/j.pt.2014.08.007. 25246022 10.1016/j.pt.2014.08.007

[53] Drake J, Carey T. Seasonality and changing prevalence of common canine gastrointestinal nematodes in the USA. Parasit Vectors. 2019;12:430. 31488192 10.1186/s13071-019-3701-7 PMC6728981

[54] Barrera JP, Miró G, Carmena D, Foncubierta C, Sarquis J, Marino V, et al. Enhancing diagnostic accuracy: direct immunofluorescence assay as the gold standard for detecting *Giardia duodenalis* and *Cryptosporidium* spp. in canine and feline fecal samples. BMC Vet Res. 2024;20:445. 39358726 10.1186/s12917-024-04297-0 PMC11445881

[55] Barrera JP, Montoya A, Marino V, Sarquis J, Checa R, Miró G. *Cystoisospora* spp. infection at a dog breeding facility in the Madrid region: infection rate and clinical management based on toltrazuril metaphylaxis. Vet Parasitol Reg Stud Reports. 2024;48:100971. 38316499 10.1016/j.vprsr.2023.100971

[56] Daba M, Naramo M, Haile G. Current status of *Ancylostoma* species in domestic and wild animals and their zoonotic implication: review. Anim Vet Sci. 2021;9:107. 10.11648/j.avs.20210904.14.

